# Determinants of livelihood diversification in rural rain-fed region of Pakistan: evidence from fractional multinomial logit (FMLOGIT) estimation

**DOI:** 10.1007/s11356-022-23040-6

**Published:** 2022-09-20

**Authors:** Nusrat Habib, Peter Rankin, Mohammad Alauddin, Rob Cramb

**Affiliations:** 1 School of Agriculture and Food Sciences, The University of Queensland, Brisbane, 4072 Australia; 2 Institute for Social Science Research, The University of Queensland, Brisbane, 4072 Australia; 3 School of Economics, The University of Queensland, Brisbane, 4072 Australia

**Keywords:** Livelihood capitals, Livelihood diversification strategies, Fractional multinomial logit, Pakistan

## Abstract

Sustainable livelihoods in less developed countries are threatened by human, natural, physical, social and financial factors. Pakistan is also facing severe negative impacts of these factors in the form of climate shocks, market imperfections and insufficient formal credit availability on rural livelihoods. This study explores rural Pakistani’s adaptation to these threats by diversifying income sources and explores the determining factors for adopting specific livelihood diversification strategies. The study is based on a quantitative survey of 295 households in three districts of rain-fed rural regions of Pakistan’s Punjab with differing annual rainfall. Results showed that households mitigated against threats to their livelihood by having a diversity of income sources (Simpson Diversity Index = 0.61). Moreover, fractional multinomial regression modelling revealed that greater education was associated with a more diversified livelihood strategy, where income was predominantly derived from off-farm and non-farm livelihood activities. On the other hand, households with older members, more livestock and larger farm size focused their livelihoods on their own farms, or primarily diversified into an off-farm strategy by working on other farms. These findings underscore the importance of improved access to education and infrastructure for livelihood diversification. A policy that focuses on reducing low literacy rates in rural Pakistan may also provide new avenues of livelihood diversifications with enhancement of rural literacy rate to mitigate the risks associated with livelihood strategies of smallholders.

## Introduction

Livelihoods are the portfolio of activities carried by households using their ability and capital to make a standard of living. Livelihood capital, on the other hand, refers to the human, social, natural, physical and financial resources crucial to the subsistence of people in response to shocks and stresses, without conceding the base of natural resources (Ansoms and McKay 2010; Ellis 2000; Iiyama et al. 2008; Habib 2021). Livelihoods are both the activities that define how people can live and the resources that ensure their standard of living (Mutenje et al. 2010). Livelihood capital can be transferred, exchanged and stored during income generation activities for an individual or for the household (Walelign 2017; Walelign et al. 2016). Expanding this terminology, livelihood strategies are the combination of choices and activities combined with the purpose to attain livelihood goals and assets, involving but not restricted to financing and production of investment strategies (Liu et al. 2018; Wang et al. 2015). However, livelihood strategies may change frequently based on the asset patterns and financial and climatic hazards faced by individuals or households (Mutenje et al. 2010). Climate change is one current threat to asset portfolios and livelihood strategies and is known as a global danger, and worldwide nations are considering urgent measures to adapt and mitigate the effects of climate change (Habib 2021; Yaro 2013). Climatic change has appeared as a warning to natural resource base and rural livelihood systems (Islam 2018), suggesting changes in livelihood strategies may be required.

Agriculture is considered as prime source of rural livelihoods and national economic growth of Pakistan, as two-thirds of the country’s residents and 80% of the poor reside and earn their livelihoods in rural areas within farming sector directly or indirectly. Agriculture is an important basis for prosperous livelihoods and an effective engine for growth of most agriculture-based countries. Unfortunately, several constraints and issues that impact Pakistan’s developmental struggles, specifically in the rural areas, adversely affect the country’s agriculture sector (GOP 2018; Hamid and Afzal 2013). However, Pakistan’s rural areas have begun to shift beyond being an economy solely based on farming (Hussain et al. 2011). Pakistan is considered as a semi-arid and arid region in the world as 80% of its land falls under these two categories. Agriculture in these areas is characterized as rain-fed with risk from variation in climatic conditions (Yousaf 2007). Therefore, agricultural productivity is vulnerable to drought, erratic rainfall and other extreme climatic conditions (Ashraf 2013; Grünenfelder 2013). The exposure of rain-fed agriculture to extreme climatic events results in considerable income risk for rural farm households (Arif 2007; Khan et al. 2017; Habib et al. 2015). The livelihoods of smallholder farmers are specifically vulnerable to climate change in Pakistan due to these agro-climatic conditions, vast dependence on rain-fed agriculture and limited capacity to diversify livelihoods (Ashraf 2013). Given the low agricultural productivity of the area and its risk to climatic change, it is important to understand the extent of livelihood diversification strategies in an area and facilitators of diversification.

World literature has informed on the dynamics of livelihood diversification in connection to livelihood assets, income strategies and poverty (Ansoms and McKay 2010; Walelign et al. 2016). Over a theoretical discussion, these studies have found that household structure, livelihood assets (human, natural, financial, social and physical), labour equalities and ecological policies are the key drivers of households’ choice of livelihood approach (Ansoms and McKay 2010; Iiyama et al. 2008; Mutenje et al. 2010). In spite of the plenty of research on the correlation between farmers’ livelihood capital and livelihood diversification strategies (Ansoms and McKay 2010; Iiyama et al. 2008; Mariam 2014; Mutenje et al. 2010; Peng et al. 2017; Waha et al. 2018; Walelign et al. 2016), the connection between livelihood capital and diversification in Pakistan’s context of semi-arid conditions and the consequent implications for local policy and livelihood attainment have not been investigated. Additionally, relative to other relevant farming areas, Pakistan has a unique livelihood asset base. Therefore, contextual research conclusions are needed to provide rational and pertinent recommendations for the predominant situations in these semi-arid areas and suggest that the precise association between livelihood capitals and the choices of livelihood diversification strategies in Pakistan’s semi-arid region is due detailed attention. Thus, this research sought to address this knowledge gap and fulfil the need for a thorough community level study of livelihood diversification strategies relative to livelihood capitals to direct policy interests and work towards sustainability of natural environment.

The primary contribution of the study, therefore, is to understand the extent of livelihood diversification and its determining factors in rural Pakistan using household surveys. The two specific questions that this paper addresses are as follows: (1) What are the current strategies utilized in terms of on-farm, off-farm and non-farm livelihoods? (2) What drives the choices of livelihood diversification? The paper is organized as follows: the next section provides a brief overview of methodology, including a study area description, data collection and analysis techniques. Thereafter, the results are presented and discussed, followed by conclusions.

## Material and methods

### Study area overview

**Fig. 1:**
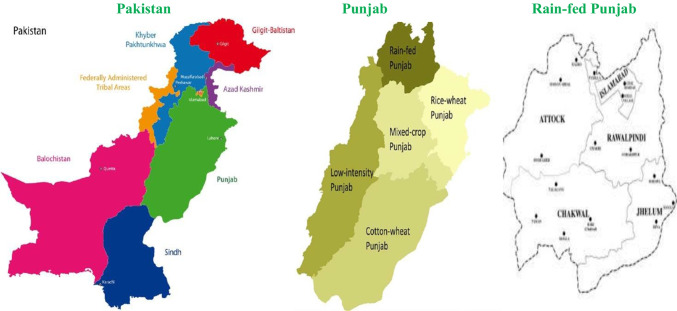
Map of the study locations in rain-fed Punjab, adapted from Khan. (2002)

The Punjab area in Pakistan can be divided into five zones based on geographical location, market access, agro-ecological potential, household activity and socio-demographic structure (Fig. 1). The study was conducted in the northern part of Pakistan’s’ Punjab that is characterized as rain-fed and known as the Pothwar plateau. It is about 250 km long and 100 km wide with elevations ranging from 200 m along the River Indus to about 900 m in the hills north of Islamabad, with an average elevation of 457 m (Khan. 2002). Rain-fed Punjab comprises the districts of Attock, Chakwal and Rawalpindi. Each of these districts was focused on in this study to represent the diversity of livelihoods in rain-fed Punjab and vulnerabilities to climate change. The selected locations are semi-arid with erratic rainfall (Ashraf et al. 1999; GOP 2009; Habib et al. 2018). Only 10% of cultivated land is irrigated, while 90% is rain-fed agriculture (GOP 2009).

### Data collection

Research data was collected by randomised household surveys. The survey used structured questionnaires to explore smallholder livelihood asset combinations (financial, social, natural, human and physical capital) and factors that may influence diversity livelihood strategies. A multistage data sampling technique was applied to sample smallholders’ households (*n* = 295). Deliberate sampling method was first employed to select three districts (Attock, Chakwal and Rawalpindi) in the rain-fed Punjab region based on high (> 750 mm), medium (500–750 mm) and low (< 500 mm) rainfall patterns. Random sample method was applied to select villages/households in each of the three selected districts. From March to May 2021, 305 households were interviewed across the study locations leading to 295 valid questionnaires (Table 1). To obtain a diverse sample, an equal number of respondents were interviewed in terms of gender (male, *n* = 148; female, *n* = 147) and districts (Table 1). Sampled households were randomly selected from different livelihood groups prevailing in the study locations based on their earning income from farm, off-farm and non-farm livelihood sources. We were interested in achieving a minimum ratio of respondents to independent variables of 10:1 (Hair et al. 2014) or 160 respondents for the 16 variables. Applying the extent of our resources and to achieve the most accuracy, we were able to approximately double this minimum and sample 295 households. The survey covered the thematic scopes of smallholders’ farmer livelihood strategies including demographics, access to and control over livelihood capital, housing facilities, perception of risk in the agriculture sector, adaptive capacity, perceived climate change resilience and gender roles and relations

**Table 1:** Study locations

District	Frequency	Per cent
Attock	100	33.90
Chakwal	97	32.88
Rawalpindi	98	33.22
Total	295	100.00

Household survey, 2021

### Proportional share of livelihood strategies

Rural households manage to engage in a portfolio of activities to diversify their sources, rather than depending upon a single source of activity for their livelihood earnings (Barrett et al. 2001; de Janvry and Sadoulet 2001; Dercon and Krishnan 1996; Ellis 1998; Ellis 1999; Rahut and Scharf 2012); hence, it is crucial to examine livelihood portfolios rather than single livelihood activity. Therefore, the study classified the livelihood strategies of rural Pakistan households into three different proportional shares:

- D1. On-farm includes those households that diversify their income source within agriculture by rotating and replacing crops on their farms to earn their livelihood.
- D2. Off-farm includes households dependent on off-farm activities to earn their livelihoods such as participating as a labourer to other farms or performing packaging, processing, storage and marketing activities of agricultural produced.
- D3. Non-farm predominately earn income from non-farm activities (such as from employment in government or non-government sectors, businesses or remittances). It is also important to mention that selection of the respondent was based on smallholders (≤ 5 acres of land), and in this non-farm category, the respondent’s dependence was on non-farm sector due to the uncertain nature of climate change. As in the study area, farming dependence was on rainfall pattern, and for these respondents, their land was left fallow from the last 2 years due to insufficient rain availability for growing any crop. Therefore, instead of having their own land, they left only to join non-farm sector to satisfy their household needs.

The frequency distribution of household livelihood types that is based on their income share earned from each category is presented in Table 2. The major type of livelihoods with percentage share was D2 (48.81%) followed by D1 (31.19%) and D3 (20%) respectively (Table 2).

**Table 2:** Livelihood proportions

Livelihood source	Frequency	Per cent
D1. On-farm	92	31.19
D2. Off-farm	144	48.81
D3. Non-farm	59	20.00
Total	295	100

Household survey, 2021

### Simpson index for measuring household diversity of livelihoods

Several indices and indicators, such as Herfindahl Index, Simpson Index, Entropy Index, Ogive index, Herfindahl–Hirschman index and the Composite Entropy Index, have been utilized to evaluate the diversification of households’ strategies. However, the Simpson Index is most frequently employed for its straightforward calculation method and wide adequacy. This study also adopts the Simpson Index from Dilruba and Roy (2012) for measuring livelihood diversification. The equation of the index is as below:

$$SI\mathrm{D}=1-\sum_{\mathrm{i}=1}^{\mathrm{N}}{\mathrm{P}}_{{\mathrm{i}}^2}$$

where *N* indicates the total number of livelihood income sources, and *P*_*i*_ denotes the fraction of the *i*th income of a livelihood source. The SID value remains between 0 and 1. When SID value is 0, this depicts an existence of specialisation, and when it approaches towards 1, there is an expansion of livelihood diversification.

### Model estimation

Econometric and statistical analysis was applied to analyse the collected survey data, using Stata-16 software (StataCorp. 2019). Descriptive statistics were calculated for each variable to examine the socio-economic status of respondents and understand the distributions of dependent and independent variables.

The primary outcome variable was the proportion of income derived from the livelihood categories of on-farm, off-farm, and non-farm source. To model changes in the three proportions as a factor of the explanatory covariates, the study used fractional multinomial logistic regression (FMLOGIT) (Mullahy 2015; Papke and Wooldridge 1996). The application of the fractional multinomial logit model in STATA software is supplied by Buis (2008). The following formula describes the model:

1 $$E\left[{y}_{ij\left|{x}_i\right.}\right]=\Lambda \left({x}_i{\beta}_j\right)=\frac{\exp \left({x}_i{\beta}_j\right)}{\left[\sum jh=1\ \exp \left({x}_i{\beta}_h\right)\right]}$$

where *y*_*ij*_ is the proportion of income from the *j*th source for the *i*th household (*j* = 1…*J*); *x*_*i*_ are the explanatory covariates (assets) for the *i*th household; and *β* is a vector of regression coefficients. The model indicates the confined nature of each proportion (i.e. 0 ≤ *y*_*ij*_ ≤ 1 for *j* = 1...*J*) as well as the fact that proportions add up to unity (i.e. ∑*Jj* = 1*y*_*ij*_ = 1∑*j* = 1*Jy*_*ij*_ = 1). It entails that the expected proportions from the model should also remain between 0 and 1 (i.e. *E* [*y*_*ij*_ | *x*_*i*_] ∈ (0,1) for *j* = 1…*J*) and add up to one (i.e. ∑*Jj* = 1*E*[*y*_*ij*_ | *x*_*i*_] = 1∑*j* = 1*JE*[*y*_*ij*_|*x*_*i*_] = 1).

Following Murteira and Ramalho (2013) and Mullahy (2011), the conditional mean evaluation of all the proportions jointly is built on the quasi-maximum likelihood estimator for this type of multinomial model condition. A contribution of a particular household to the likelihood is:

2 $${L}_i\left(\beta \right)={\prod}_{j=1}^JE\left\{{y}_{ij}|{x}_i\right\}{y}_{ij}$$

The sum of a particular household log-likelihoods is maximised to achieve the estimator for *β*:

3 $$\overset\vee\beta=\max\nolimits_\beta\sum\nolimits_{i=0}^N\;Log\;L_{i\left(\beta\right)}$$

Fractional multinomial models are the preferred model when the true data generation process is fractions of three or more choices that sum to 1 and can include real 1s and 0s where a single choice dominates all others (Murteira and Ramalho 2013).

### Livelihood assets and research hypothesis

The sustainable livelihood framework (SLF) was applied to explore the impacts of livelihood assets in determining livelihood diversification. In SLF livelihood, assets represent the resources possessed by an individual or by a household that are classified into five categories: (1) human capitals (education, skills), (2) financial capital (savings, formal credit availability), (3) natural capital (land, orchard, water), (4) social capital (social linkage or networks), and (5) physical capital (infrastructure). This method considers the asset position of the smallholders as fundamental to knowing the choices open to them, the strategies they can adopt for maintaining their standard of living or survival (Allison and Ellis 2001; Smith et al. 2005). The hypothesis of this study is bidirectional as the data for this study is collected at one time point; therefore, we have decided to test the hypothesis that livelihood capitals in various forms are associated with livelihood diversification strategies. This study employed the following asset classification (Table 3) as explanatory variables in the regression models.

**Table 3:** Livelihood assets employed in multivariate analysis

Human	Natural	Physical	Social	Financial
Total number of households	Total farm area in acres	Access to road	Leadership quality	Access to credit
Education of respondent in years	Total number of livestock	Distance from market	Access to extension service	Who make final decision about taking loan/credit? (Dummy: 1 = male, 2 = female)
Age (years)		Access to public transport		
Total family labour		Technological advancement		
Gender (male or female)				

## Results

### Socio-demographic and livelihood characteristics

Descriptive analyses for key socio-demographic and livelihood characteristics variable of the study respondents are presented in Table 4. The respondents’ age, education level, gender, household size, marital status, sources of income and livelihood and overall diversity index were key features of this study. The majority (49.15%) of respondents were young, within the age range of 18–30 years, followed by a medium age group ranging 31 to 50 years (36.95%). The average age of the respondents in the overall sample was 35.26 years. Most respondents were educated (53.22%), with 30.85% respondents uneducated who cannot read or write. Average household size was 6.64. Almost 70% of the respondents were married. Livelihood sources of income in the study area were D1 (on-farm), D2 (off-farm) and D3 (non-farm) combinations, with half of the sample households’ livelihood source from D2 (off-farm). Finally, a high positive Simpson diversity index (0.63) illustrated that in rural Pakistan, there is a diversity of livelihoods aside from farming.

**Table 4:** Socio-demographic and livelihood profile of respondents

Characteristics	Frequency (%)
Age (years)	
18–30	145 (49.15)
31–50	109 (36.95)
51–75	41 (13.90)
Mean	35.26
Education of the respondent	
Uneducated	91(30.85)
Secondary (high school) diploma or equivalent certificate or college level	157 (53.22)
University level	47 (15.93)
Gender	
Male	148 (50.20)
Female	147 (49.80)
Household size	
1–4	53 (17.96)
5–8	179 (60.68)
Above 9	63 (21.36)
Mean	6.64
Marital status	
Married	206 (69.83)
Single	76 (25.76)
Divorced/widowed	13 (4.41)
Livelihood sources	
D1: On-farm	92 (31.19)
D2: Off-farm	144 (48.81)
D3: Non-farm	59 (20.00)
Simpson income diversity index (SID)	0.63

Household survey, 2021

### Multivariate analysis

Fractional multinomial regression modelling was used to analyse how livelihood capitals were associated with different livelihood strategies for smallholders in rural Pakistan. The estimation results are shown in Table 5. The livelihood strategies D1 (on-farm), D2 (off-farm) and D3 (non-farm) were used in this analysis. The D3 (non-farm) category was used as base category for estimation. These results, however, are not directly used to evaluate the magnitude and sign of the coefficients. For this purpose, marginal effects are calculated, and the results of the marginal effects analysis with significance levels are shown in Table 6. The marginal effects are explained as the projected percentage point change (in hundredths) in the share of a certain strategy of households’ livelihoods. The dependent variable is the proportion of income each household receives from the three livelihood categories.

**Table 5:** Fractional multinomial logit model (FMLOGIT) estimates for determinants of livelihood strategies

Determining factors	On-farm proportion (D1)		Off-farm proportion (D2)	
	Coefficients	St. Error	Coefficients	St. Error
Human capital				
Household size	0.2239***	0.1031	0.2453**	0.1107
Education (secondary/high school)	− 2.2373***	0.4460	− 1.4134***	0.4833
Education (University level)	− 3.4041***	0.4244	− 2.9641***	0.4584
Age	0.0415***	0.0138	0.0261	0.0146
Labour force	0.5729***	0.1504	.4752***	0.1563
Gender (female)	− 0.2703	0.2917	0.9692	0.3110
Natural capital				
Farmland	0.3223***	0.0965	0.2637	0.0975
Livestock size	− 0.0480	0.0769	− 0.1788	0.0818
Physical capital				
Access to road	− 0.0123	0.0230	− 0.0038	0.0261
Distance from market	− 0.0060	0.0136	− 0.0066	0.0139
Access to public transport	0.0800	0.4176	− 0.4896	0.5023
Technological advancement	0.5243	0.3213	− 0.4523	0.3508
Social capital				
Leadership quality	1.4106	1.178	− 0.2782	0.8024
Access to extension service	− 0.0061	0.0099	− 0.0080	0.0109
Financial capital				
Access to credit	− 0.0308	0.5697	0.0633	0.5661
Who decides (female)	− 0.0716	0.2394	− 0.0535	0.2362
Constant	− 5.1740	2.5966	0.4556	2.0203

*n* = 295. Log pseudo-likelihood = − 226.76354

***Significance at 0.01% level; **significance at 1% level; *significance at 5% level

**Table 6:** Marginal effects of variables included in the FMLOGIT model

Determining factors	On-farm proportion (D1)		Off-farm proportion (D2)		Non-farm proportion (D3)	
	Coefficients	St. Error	Coefficients	St. Error	Coefficients	St. Error
Human capital						
Household size	0.0011	0.0103	0.0141	0.0122	− 0.0152**	0.0068
Education (secondary/high school)	− 0.2156***	0.0462	0.1418***	0.0503	0.0737***	0.0175
Education (university level)	− 0.2159***	0.0617	− 0.0584	0.0753	0.2743***	0.0527
Age	0.0041**	0.0015	− 0.0021	0.0017	− 0.0020**	0.0009
Labour force	0.0340**	0.0139	− 0.0010	0.0159	− 0.0329***	0.0095
Gender (Female)	− 0.2535***	0.0381	0.2841***	0.0422	− 0.0306	0.0189
Natural capital						
Farmland	0.0198	0.0137	− 0.0014	0.0145	− 0.0183***	0.0060
Livestock size	0.0255**	0.0096	− 0.0338***	0.0108	0.0082	0.0050
Physical capital						
Access to road	− 0.0020	0.0018	0.0015	0.0025	0.0004	0.0050
Distance to nearest market	− 0.0000	0.0017	− 0.0003	0.0018	0.0004	0.0008
Access to public transport	0.1185*	0.0638	− 0.1360	0.0752	0.0174	0.0287
Technological advancement	0.2126***	0.0529	− 0.2177***	0.0580	0.0051	0.0204
Social capital						
Leadership quality	0.3801*	0.2053	− 0.3565*	0.1873	− 0.0235	0.0547
Access to extension service	0.0002	0.0012	− 0.0007	0.0014	0.0004	0.0006
Financial capital						
Access to credit	− 0.0200	0.1006	0.0217	0.1050	− 0.0017	0.0335
Who decides/female	− 0.0054	0.0381	0.0015	0.0397	0.0038	0.0142

***significance at 0.01% level; **significance at 1% level; *significance at 5% level

The explanatory variables constituted a livelihood asset pentagon (human, natural, physical, social and financial capital) that may influence a households’ decision to diversify their livelihood strategies.

#### Human capital

The analysis of marginal effects showed that human capital was the most important determining force in livelihood diversification process, as all of the selected independent variables of human capital significantly affected the adoption of livelihood strategies. Specifically, as household size increased, the proportion of D3 (non-farm) income decreased by 1.52%. Contrary to prior expectation, a unit increase in years of age for the respondent was associated with an increased proportion of D1 (on-farm) by 0.41%, whilst increases in age were associated with a decrease in the proportion of D3 (non-farm) income by 0.20%. This may be because young family members are comparatively well educated and have more access to technologies, and therefore, they look to, and take risks in, non-farm livelihood opportunities.

As expected, education level was one of the greatest significant determinants of livelihood diversification. Education level had a positive association with adopting D2 (off-farm) and D3 (non-farm) livelihood strategies. The highly educated households diversified their livelihood choices via remunerated jobs, self-employment, trading or in off-farm activities, whereas the less formally educated households involve themselves in lower-priced labour, less wage earnings and less non-farm activities. More educated households have additional knowledge and skill that allow them greater opportunity to perform non-farm activities, than illiterate and poorly educated households. Compared to those without secondary education, those with secondary/high school education had a greater proportion of D2 (14.18%; off-farm) and D3 (7.37%; non-farm) income and 21.56% less D1 (on-farm) income. However, those with university level education only had a larger proportion of D3 (27.43%; non-farm) income and less on-farm income (21.59% lower D1).

Family labour force was associated with a greater proportion of D1 (on-farm) income and a lower proportion of D3 (non-farm) income. Specifically, a unit increase in the size of the family labour force was associated with a 3.40% increase in proportion of D1 (on-farm) income and a 3.29% decrease in the proportion of D3 (non-farm) income. Female household had a lower proportion of D1 (on-farm) income and undertook a greater proportion of D2 (off-farm) activities. This may be because farming is typically dominated by male household members who are the landowners and decision makers.

#### Natural and physical capital

A unit increase in livestock size was positively associated with D1 (on-farm) income and negatively with off-farm income. Respondents with more livestock derived a greater proportion of income from D1 (on-farm) activities, and, in contrast to expectations, a 3.38% lower proportion of income from off-farm activities. On the other hand, a unit increase in farmland was associated with a 1.83% lower proportion of D3 (non-farm) income. Access to public transport and technological advancement was positively and statistically significantly linked with a greater proportion of income from D1 (on-farm) sources, 11.85% and 21.26% respectively. On the other hand, technological advancement was associated with a lower proportion of income from off-farm activities, suggesting households without advanced physical capital need to derive more income from off-farm sources.

#### Social capital

Those households with strong social networking via the leadership quality of the respondent derived a 38.01% greater proportion of income from D1 (on-farm) sources, whilst those with leadership quality obtained 35.65% less of their income from D2 (off-farm) activities. Access to extension services performs a main role in attaining and improving the rural development goals, but was not significantly associated with the proportion of income derived from the three livelihood activity categories. The potential justification for this could be that extension workers are also providing entrepreneurial skills to farmers instead of only offering agricultural extension services; this could be an entry point for joining of non-farm business strategies.

#### Financial capital

The estimates for the role of financial capital revealed no statistically significant links between access to credit and finical decision-making by women and the three livelihood categories. This may be because smallholder households in rural Pakistan in general have limited access to formal credit, because banking sectors prefer large landholders when granting formal loans.

## Discussion

This study investigated the association between livelihood capital and the proportional share of livelihoods’ diversification strategies for smallholders in rural Pakistan. Before exploring the major determining factors of livelihood diversification strategies, the study sorted three proportional shares of livelihood strategies: D1 (on-farm), D2 (off-farm) and D3 (non-farm) and estimated the diversity index for selected sample (SID = 0.63) that depicts the existence of substantial diversification in the study area. This finding of diversity index is consistent with research in other less-developed countries (Adem et al. 2018; Agyeman et al. 2014; Schwarze and Zeller 2005). In rural communities, due to scare resources, there is a common and imperative need for smallholders to adopt livelihood diversification either within farming or into non-farm activities that may offer greater livelihood security (Asfaw et al. 2017; Dapilah et al. 2020). Households that diversified their livelihood either within the farming or into non-farm sectors are afforded the best prospect of being resistant to natural shocks, rather than relying on only traditional farming practices (Adem et al. 2018). Our findings coincide with Babatunde (2013) and Pfeiffer et al. (2009), who establish that livelihood diversification in farming and non-farming sectors helps balance resources for both the sectors and can provide financial support to each other in times of need (Abdallah 2016; Twumasi et al. 2019). Non-farm activities can provide financial support to farming sector both in short-term (purchase of seed, fertilizer) and for long-term purposes, e.g. investment in tunnel farming set up (Peng et al. 2017; Pfeiffer et al. 2009). For long-term security, ideal diversification would include a mix of different strategies in the form of on-farm, off-farm and non-farm that can enhance adaptation capacity in adverse conditions or at times of any natural disaster (Allison and Ellis 2001; Barrett et al. 2001; Mukadasi 2018; Peng et al. 2017; Pfeiffer et al. 2009; Walelign et al. 2016).

This study filled a gap in knowledge by studying the role of livelihood capital in shaping livelihood strategies pursued by smallholder communities. Despite exploring the overall link between livelihood assets and livelihood diversification (Haggblade et al. 2010), the role that livelihoods capital can play in adopting diverse portfolio of strategies as an adaptation to environmental and social shocks has not been fully explored. Some studies have assumed that livelihood diversification is beneficial only when it focuses on non-farm income sources (Barrett et al. 2001; Dapilah et al. 2020; Haggblade et al. 2010; Tsiboe et al. 2016). But this study offers an empirical narrative that smallholders’ community adopts various proportions of livelihood strategies in the form of D1 (on-farm), D2 (off-farm) and D3 (non-farm) that can be helpful in facilitating the resources and improving the overall livelihood of a household. This proportional share combination can be beneficial for inflow of capital between strategies, especially when one sector is in need, particularly in resource-limited settings like semi-arid Pakistan and may prove beneficial for smallholders in other part of the developing world (Yaro 2013). The results of this study indicate that households in D1 (on-farm) sector are diversifying their livelihoods within the farming sector that can be assumed as changing crop varieties or altering and adopting multiple cropping patterns as a common livelihood approach for them. These findings are consistent with Beets (2019), Mukadasi (2018) and Waha et al. (2018) who indicate that multiple cropping patterns provide safeguard and security against natural disasters to earn a stable household income.

We identified several associations between human capital and different proportionate shares of livelihood strategies, though previous studies present mixed findings about these associations. Breman (1996) conducted a study in in India and found similar results, in that younger households adopted more livelihood strategies in the non-farm sector compared to older households. Kimhi and Lee (1996) suggested a nonlinear relationship between age and livelihood diversification patterns. On the other hand, Barrett et al. (2001) and Web (2001) discussed that older household heads have a larger family size and are likely to have extra labour that helps them allocate time outside the agricultural sector. Education is considered an important determinant of livelihood diversification by many researchers (Abdulai and CroleRees 2001; Barrett et al. 2005; Canagarajah et al. 2001; Ellis 2000; Escobal 2001; Lanjouw and Feder 2001; Lanjouw and Shariff 2004; Micevska and Rahut 2008; Reardon 1997).

The results of this study revealed that respondents who attended greater formal educational training had a higher proportion of livelihood diversification into non-farm and off-farm livelihood shares compared to those who do not have any formal education. Existing literature has also found a similar, positive association between education and diversification (Barrett et al. 2001; Gebre-Egziabher et al. 2000; Kimhi and Lee 1996), whilst Woldehanna (2000) reported that education adversely affects on-farm sector. This was qualified, however, by noting that further education had a strong positive association with non-farm livelihood success. Ellis (1998) in his theoretical discussion articulated that livelihood diversification strategies that involved higher skilled labour force attract more educated households, and strategies that involved lower skilled labour attract less or un-educated household members of a society.

Natural capital comprises some key indicators like farmland and number of livestock owned by a household (Baird and Hartter 2017; Sarker et al. 2019). The number of livestock was positively associated with the proportion of D1 (on-farm) income, with similar findings found by Islam (2018) who conducted a study in Bangladesh. Physical capital provides a sound ground for diversifying income sources in developing countries (Barrett et al. 2001; Clarke and Carney 2008; Mallick 2019; Nguyen et al. 2015); however, in this study, physical capital was associated with a larger proportion of on-farm income, suggesting those with greater physical capital relied on their farm in comparison to being indicative of diversification. Organizational participation constituting social capital is considered as a significant contributor for diversification (Paul and Islam 2015). Our study, however, presents contrary findings in that the households who have opportunity to participate in social organizational activities received a greater proportion of income from on-farm sources. The analysis indicated that access to extension services was not significantly associated with livelihood diversification choices, which stands in contrast to existing literature (Asfaw et al. 2017; Gebreyesus 2016; Sarah 2019). Sarker et al. (2019) mentions the importance of financial capital for diversification, but significant associations were not identified in this study. The reason for this could be that smallholders have less access to formal loans or face hurdles in getting loans from banks. In comparison to existing literature, this study provides additional knowledge and insights in defining the determining forces for livelihood diversification in rural Pakistan.

## Conclusions and policy implications

This study was conducted in the rain-fed region of Pakistan where rural livelihoods are increasingly exposed to natural, social and financial vulnerabilities in the form of climate change, lack of networking and formal credit availability. This study applied SLF analysis to determine and expose the factors of livelihood diversification in three selected districts of Pakistan (Attock, Chakwal and Rawalpindi). The study found that smallholder farmers in Pakistan’s Punjab use diverse livelihood strategies to achieve livelihood objectives and sustain their households. The overall diversity index (0.63) also indicates that rural household earn their livelihoods from diversified strategies instead of remaining only within the farming sector (D1). The major livelihood source was an off-farm strategy (D2). Furthermore, the fractional logistic modelling demonstrated that the human, natural, physical and social assets of households have strong association with the choice and adoption of a specific proportional share of livelihood diversification strategies in the study area. From the livelihood asset pentagon, the number of household members, education, age, farmland, size of livestock, technological advancement and leadership roles were the major determining factors of the proportional share of income from each of the livelihood strategies.

Based on the findings, to improve and develop the strategies of livelihood diversification and enhance overall livelihood status of smallholders, the study provides the following recommendations and policy implications:

1. The government could invest in the provision of free and quality education to rural communities. Providing free access to better education will help them to adopt and contribute in non-farm and off-farm income earning livelihood strategies.
2. The government could support and acknowledge non-farm livelihood diversification strategies as a part of nationwide job construction objective, instead of only subsidizing inputs for agriculture sector.
3. The expansion of roads to connect rural-urban markets can play a vital role in accessibility and adoption of non-farm livelihood income sources for rural communities.

## Theoretical, practical and societal implications

The findings of the study provide a realistic insight into the livelihood income creating approaches used at the micro household level and highlight how rural populations diversify their livelihood strategies to achieve better economic outcomes within farming or non-farming sectors. Rural diversification not only determines household asset allocation, but also determines household labour resource allocation in numerous livelihood activities. As Pakistan has a large number of rural youth, a comprehensive understanding of rural labour markets and the diversified sources of income and livelihood strategies would assist in the design of rural and urban developmental policies.

## Study limitations and future research areas

Although we randomized study participation, stratified our sample to include multiple localities and used advanced statistical techniques to evaluate livelihood allocation, several limitations are notable. First, surveys were limited to a subset of the population and may not have captured all livelihood approaches. Additionally, respondents may have made errors in recalling information and specifying proportional sources of income. The sample was predominately from farming communities with high rates or illiteracy and low levels of formal education, so records were not available to validate the output of their livelihood activities. This study was also impacted by the COVID-19 pandemic in two ways: (1) data collection cost doubled and reduced our sample due to necessary field protocols, such as providing masks/hand sanitizer to respondents in the community, and (2) the study took longer than our estimated number of days that cost us extra in terms of travelling cost. Important avenues for future research areas include (1) exploring the impact of agricultural subsidies on farm-based livelihood strategies recently introduced in Pakistan; (2) understanding the impact of diversification on gender equality in access to basic services such as health, education, and employment (3) and evaluating the role of agricultural extension services to providing services that improve rural livelihoods.
